# A dataset for fatigue estimation during shoulder internal and external rotation movements using wearables

**DOI:** 10.1038/s41597-024-03254-8

**Published:** 2024-04-27

**Authors:** Merve Nur Yasar, Marco Sica, Brendan O’Flynn, Salvatore Tedesco, Matteo Menolotto

**Affiliations:** Tyndall National Institute, University College Cork, Cork, T12 R5CP Ireland

**Keywords:** Biomedical engineering, Research data

## Abstract

Wearable sensors have recently been extensively used in sports science, physical rehabilitation, and industry providing feedback on physical fatigue. Information obtained from wearable sensors can be analyzed by predictive analytics methods, such as machine learning algorithms, to determine fatigue during shoulder joint movements, which have complex biomechanics. The presented dataset aims to provide data collected via wearable sensors during a fatigue protocol involving dynamic shoulder internal rotation (IR) and external rotation (ER) movements. Thirty-four healthy subjects performed shoulder IR and ER movements with different percentages of maximal voluntary isometric contraction (MVIC) force until they reached the maximal exertion. The dataset includes demographic information, anthropometric measurements, MVIC force measurements, and digital data captured via surface electromyography, inertial measurement unit, and photoplethysmography, as well as self-reported assessments using the Borg rating scale of perceived exertion and the Karolinska sleepiness scale. This comprehensive dataset provides valuable insights into physical fatigue assessment, allowing the development of fatigue detection/prediction algorithms and the study of human biomechanical characteristics during shoulder movements within a fatigue protocol.

## Background & Summary

Repeating the same, or comparable, motions over time can cause some muscle groups to become overused and overextended, resulting in muscular fatigue^1^. Monitoring fatigue in the shoulder joint is crucial, considering its vital role in diverse athletic activities, work-related tasks, and rehabilitation routines^2–5^. Measuring shoulder fatigue of upper extremities in sports involving repetitive and high-intensity movements, such as throwing, tennis volley and serves, golf swing, and swimming strokes, allows trainers to control athlete’s training, providing personalized sessions and diminishing injury susceptibility^6–10^. Physiotherapists can customize exercise programs to provide safe and progressive rehabilitation that balances muscle recovery and prevents overstrain by monitoring fatigue levels during shoulder rotation exercises^3,11–13^. Shoulder movements are also critical for various industrial and occupational tasks, such as lifting, assembling, reaching, pulling, and pushing during prolonged or repetitive movements^14–18^. In environments where workers are at risk of being exposed to work-related shoulder fatigue, which is a risk indicator for musculoskeletal disorders, it is essential to understand the physiological and biomechanical patterns that can induce fatigue and take precautions^15,19–21^.

Due to their ability to generate real-time data, cost-effectiveness, non-invasiveness, remote monitoring, and portability, wearable devices are frequently utilized in a variety of applications to detect or monitor physical fatigue, including medical rehabilitation^22^, sports applications^23^, and occupational health and safety^24,25^. In this context, the present work provides a comprehensive set of data to globally assess physical fatigue during tasks involving shoulder internal rotation (IR) and external rotation (ER) movements holding a series of weights corresponding to different percentages of maximal voluntary isometric contraction (MVIC) force. The dataset includes data generated by wearable surface electromyography (EMG), inertial measurement unit (IMU), and photoplethysmography (PPG), as well as the results of subjective self-reporting tests, such as the Borg rating of perceived exertion (RPE) scale and Karolinska sleepiness scale (KSS).

EMG enables continuous muscle activity monitoring, providing information on muscle contractions^26^. Physical fatigue is typically detected from EMG signal as an increase in amplitude and changes in the frequency spectrum^27,28^. By positioning EMG sensors on the involved muscle groups, time to fatigue can be measured, and muscle activation patterns can be tracked over time^29^. IMU sensors generally comprise a triaxial accelerometer (acc), triaxial gyroscope (gyr), and triaxial magnetometer (mag). These sensors can be attached to various body segments to capture orientation and motion data, providing insight into kinematic information during the physical task, and related changes caused by physical fatigue^30,31^. PPG sensors, often integrated into wearable devices, enable the non-invasive measurement of blood flow and heart rate variability, offering information about an individual’s physiological state^32,33^. By monitoring changes in PPG signals, such as pulse rate, oxygen saturation, and vascular tone, early signs of physical fatigue, stress, and decreased cognitive function can be detected^34–36^. Concerning the subjective self-reporting assessments, the Borg RPE scale assesses an individual’s exertion, strain, and fatigue during physical activity based on physical signs (such as an elevated pulse rate and muscle fatigue) experienced by the subject^37^. In addition, fatigued individuals may feel raised sleepiness and decreased ability to stay awake^38^. Some studies found a significant correlation between sleepiness and physical fatigue^39^. In the contest of our study, the KSS value offers an indication of the subject capability and reactiveness at the day the data were collected, providing an extra level of information in processing and interpreting the objective data collected by the wearables^40,41^.

Although several datasets are available for fatigue measurement with wearable devices^25,42–47^, none contain information on physical fatigue during complex repetitive shoulder joint movements with varying loads. By combining objective and subjective assessments, this dataset provides a comprehensive and adaptive tool for model fatigue, during shoulder rotations at varying levels of contraction force. Incorporating various wearable sensors to assess fatigue can aid in the development of objective detection of muscular fatigue via modelling or data-driven approaches, such as machine learning. Previous studies have integrated artificial intelligence techniques into fatigue management strategies, using wearable sensor data for physical fatigue prediction and tailor manual tasks or physical training to enhance human performance. These models have effectively monitored and detected how a person’s endurance degrades when fatigue sets in^48–50^; nevertheless, there is a need to understand how fatigue differs according to changes in people’s physical characteristics^49^. Individual features such as age, gender, fitness, and previous injuries contribute to variation in human performance^49,51^. Based on the anthropometric measurements and wearable sensors, data supplied in this dataset can be utilized to construct tailored machine learning approaches that consider physical characteristics that differ across individuals. Moreover, this database seeks to provide data for studies aiming at optimizing training regimens, injury prevention protocols, and workplace ergonomics by investigating the effects of varying MVIC force percentages on fatigue and evaluating the relationship between perceived exertion and physiological measures. As wearable EMG, IMU, and PPG sensors have proven useful for fatigue recognition, the dataset provides an open and easy-to-download resource for building and testing relevant algorithms. It can also be used by researchers interested in physical fatigue detection to compare and validate algorithms.

## Methods

The study was approved by the Clinical Research Ethics Committee (CREC) of the Cork Teaching Hospitals at the University College Cork under Reference ECM 4 (p) 6/7/2021 & ECM 3 (ww) 09/08/2022 and adhered to ethical regulations. Data were collected at the Wearable Laboratory at the Tyndall National Institute, University College Cork, in Cork, Ireland from April to July 2023.

### Participants

This study comprised 34 healthy participants (female:11, male:23, age: 26 ± 4 years) without musculoskeletal injuries. Subjects were recruited through word of mouth and subsequently detailed information about the experiment protocol was provided via e-mail. Participants were instructed to avoid excessive physical activity before the data capture. They were also cautioned not to consume stimulants (caffeine, energy drink etc.) before the measurements as stimulants can affect performance and focus^52,53^. These effects could have a positive impact on reducing fatigue and improving alertness, potentially leading to inconsistent results^54^. On the measurement day, subjects signed the informed consent to indicate their intention to join the study. Most participants were right-handed, with two individuals reporting left-handed dominance. They were in general physically active, specifically engaging in fitness training on an average of three times a week.

### Experimental protocol

Demographic and anthropometric data were initially collected for each volunteer. Later, an upper extremity dynamic warm-up protocol was conducted, including wrist flexion and extension, large and small forward and backward circles, arm taps and hugs, standing rotation, and internal and external shoulder rotations. This approximately seven-minute routine was carried out to prepare the upper extremity muscles involved in the movement physiologically and prevent a possible risk of injury^55^. Once participants completed the warm-up protocol, EMG electrodes and IMU sensors were placed on the muscles and joints on the dominant side of the upper body; positions are described in the ‘EMG measurement’ and ‘IMU measurement’ sections, respectively. Afterward, KSS was administrated to participants to determine their sleepiness states at the beginning and very end of the measurements^41^. The MVIC forces of the participants during shoulder IR and ER movements were measured using a push/pull dynamometer (Walfront NK-500). The tests were repeated twice with a two-minute interval between each repetition. After MVIC force measurement, the PPG wearable wireless sensor was placed on the non-dominant index finger to measure blood volume changes during the shoulder IR and ER tasks. Participants then performed shoulder IR and ER exercises with cable pulley apparatus with three different weight (kg) ranges corresponding to 30–40%, 40–50%, and 50–60% of their MVIC force. In order to minimize order effects, given that the proposed tasks affect participant performance differently and require different conditions (varying body posture and weight), the volunteers were divided into three groups. Each group performed the tasks in a different order, as outlined in Table 1. This pseudo-randomization contributes to meeting the statistical assumption of independence, enhancing the generalizability of the study’s findings.

**Table 1:** Task orders.

Group	Number of participants	Task order					
		I	II	III	IV	V	VI
Group one	12	IR 30–40%	IR 40–50%	IR 50–60%	ER 30–40%	ER 40–50%	ER 50–60%
Group two	10	ER 50–60%	ER 40–50%	ER 30–40%	IR 50–60%	IR 40–50%	IR 30–40%
Group three	12	IR 50–60%	ER 50–60%	IR 40–50%	ER 40–50%	IR 30–40%	ER 30–40%

**Fig. 1:**
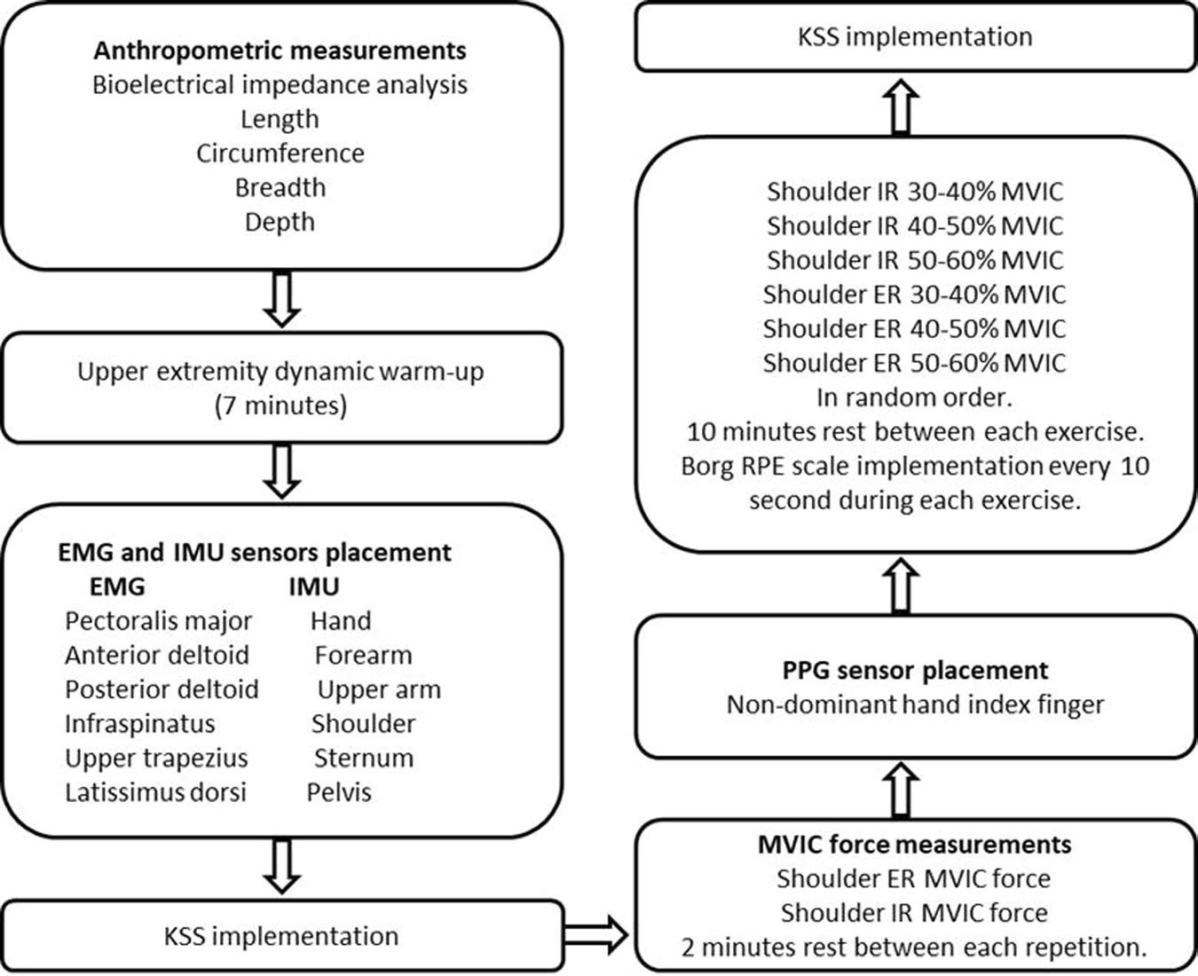
Experimental design.

The shoulder IR movement was performed while subjects were standing in a lateral position next to the fixed cable pulley. The shoulder IR movement was performed while subjects were standing in a lateral position next to the fixed cable pulley. Shoulder rotation movements under investigation are predominantly executed in a standing position in sports practices such as racket sports, rehabilitation settings for shoulder injuries, and industrial practices like pick-and-place activities, the standing position was chosen as it better reflects real-world scenarios to simulate more practical and realistic conditions^56,57^. They held the U-handle cable attachment, maintaining a neutral shoulder position with their upper arm at the side of the body. The elbow was flexed to 90 degrees, the wrist was kept straight, and the arm was rotated inward towards the abdomen. For the shoulder ER movement, the same body posture was required. Subjects remained in a standing position on the lateral side of the fixed cable pulley, holding the U-handle cable attachment. The shoulder maintained a neutral position with the upper arm alongside the body, while the elbow was flexed at a 90-degree angle, and the wrist was kept straight. In this case, the arm was rotated outward, away from the front of the body. The Borg RPE was utilized as a subjective scale for participants to determine fatigue levels before and every 10 seconds during shoulder IR and ER exercise. Participants continued to perform the task until they reached the exhausting level (20) on the Borg RPE Scale. The scale was positioned at eye level in front of the participants during each exercise and monitored. Participants were verbally instructed to maintain the required body position and continue the exercises until completely exhausted. A metronome sound recording of 40 beats per minute was used to ensure the exercises were performed uniformly at the same speed during the tests. A 10 min rest interval was given between each measurement. A step-by-step description of all the stages in the protocol and the data collected in each stage is provided in Fig. 1.

### Anthropometric measurements

Body composition - Body weight (kg), body mass index (BMI) (kg/m^2^), body fat (%), visceral fat, and skeletal muscle (%) - was determined by the clinically proven bioelectrical impedance analysis (OMRON BF511 T Monitor, Healthcare Co., Ltd. Kyoto, Japan) (https://www.omron-healthcare.com/products/bf511-turquoise) following Omron guideline^58^ (Fig. 1). A measuring tape (Bozeera Body Mass Tape 150 cm) was utilized to obtain standing height, length (upper arm, forearm, palmar, and hand) and circumference (upper arm distal (UAD), upper arm proximal (UAP), upper arm middle (UAM), upper arm tense (UAT), forearm distal (FD), forearm proximal (FP), forearm middle (FM), and hand (H) measurements (all expressed in cm). An electronic digital calliper (Neiko 01407 A USA, 0–150 mm) was used to assess breadth and depth measurements of the UAD, UAP, UAM, FD, FP, FM, and H. Measurements were taken from the dominant side of the body. Measurements were conducted using the arm reference points outlined by Neuman *et al*.^59^ and the hand reference points described by Garrett (1971)^59,60^.

### MVIC force measurement

An ad-hoc setup was prepared to perform the shoulder IR and ER MVIC force tests using the Walfront NK-500 push/pull dynamometer (Fig. 1). Firstly, the dynamometer U-shape handle and clamp were designed ad-hoc and fabricated using a fused filament modelling 3D printer. Afterward, The U-shaped holder was fastened to the dynamometer with a non-stretch rope. The dynamometer was arranged inside the clamp and positioned horizontally on a 90 cm high stable table. During the MVIC force tests, participants sat on a stable bench placed parallelly alongside the table. Their feet were flat on the ground, and their shoulders were neutral with zero abduction. They kept their upper arms on the side of their bodies while their elbows were flexed at 90 degrees. Participants were asked to stabilize their spine and avoid scapular protraction and retraction as they could compromise shoulder rotation. In the shoulder IR and ER MVIC tests, the dominant arm was employed to apply resistance using the U-shaped hand grip. In the shoulder IR MVIC test, participants were instructed to exert full force (maximum effort) while attempting to rotate their arm against the dynamometer internally. In the shoulder ER MVIC test, participants were instructed to exert full force (maximum effort) while attempting to rotate their arm against the dynamometer externally. Subjects were carefully monitored throughout each isometric test to ensure that they maintained the required shoulder position and did not attempt compensatory movements of the scapula or trunk. During the shoulder IR and ER MVIC force test, the subjects were positioned according to the direction of force application. Each MVIC force test lasted for around five seconds and was repeated twice. Two-minute rest intervals were given between each repetition. EMG and IMU sensors were recorded simultaneously during MVIC force tests.

### EMG measurement

**Fig. 2:**
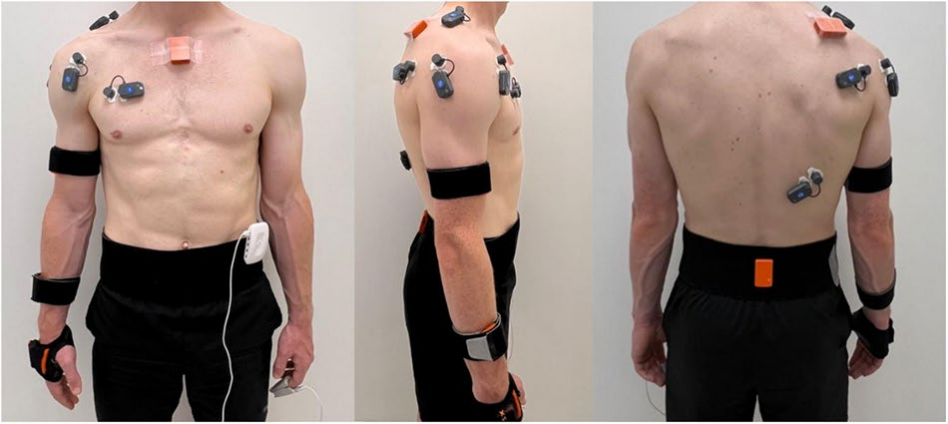
EMG, IMU, and PPG sensors placement.

Muscle electrical activity was recorded with 1000 Hz sampling frequency by surface EMG (BTS FREEEMG 300, BTS Bioengineering, Italy) (https://www.btsbioengineering.com/products/freeemg) during MVIC force measurements and shoulder IR and ER exercises (Fig. 1). Standard pre-gelled 24 mm adhesive electrodes from Covidien Kendall were positioned on the pectoralis major, anterior deltoid, infraspinatus, posterior deltoid, upper trapezius, and latissimus dorsi at the dominant side of the body. Electrodes were placed using the reference areas described in Cram’s Introduction to Surface Electromyography^61^. Every electrode weighed 13 grams and had a 16-bit resolution, along with a common mode rejection ratio (CMMR) of >110 dB at 50–60 Hz and an input impedance of 100 MOhm^62^. Before attaching the EMG electrodes, if necessary, participants’ skin was carefully shaved and cleaned with a 70% alcohol-based gel to reduce impedance between the skin and electrodes and ensure optimal signal transmission^63^. A trigger box (The FreeEMG Xsens) was used to synchronize IMU and EMG devices. EMG sensors placement is shown in Fig. 2.

### IMU measurement

IMU sensors (MTw Awinda, Xsens Technologies B.V., Enschede, The Netherlands) (https://www.movella.com/products/wearables/xsens-mtw-awinda) were used to obtain acc, gyr, and mag data at a sampling frequency of 100 Hz. These sensors collected raw data on acceleration, angular velocity, and magnetic field intensity along the x, y, and z axes for each sensor independently and synchronously^64^. Six IMU sensors (each weight: 16 gr, dimensions: 47 × 30 × 13 mm) were placed on the dominant hand, dominant forearm, dominant upper arm, shoulder, sternum, and pelvis, with elastic Velcro straps and medical tape according to the manufacturer’s instructions (https://base.movella.com/s/article/Sensor-Placement-in-Xsens-Awinda-System?language=en_US) (Fig. 1). Straps and medical tapes were fastened tightly enough to the body segments to ensure that the sensors remained fixed and immobilized. IMU sensors placement is shown in Fig. 2. Before the measurements, sensors were aligned side-by-side in a lateral position. They were then calibrated with the “heading orientation” option of the Xsens system’s acquisition software so that their orientation was correctly established with respect to testing area. After completing the measurements, the sensors were placed back in their initial side-by-side arrangement to visually confirm that there was no drift in the raw values.

### PPG measurement

**Fig. 3:**
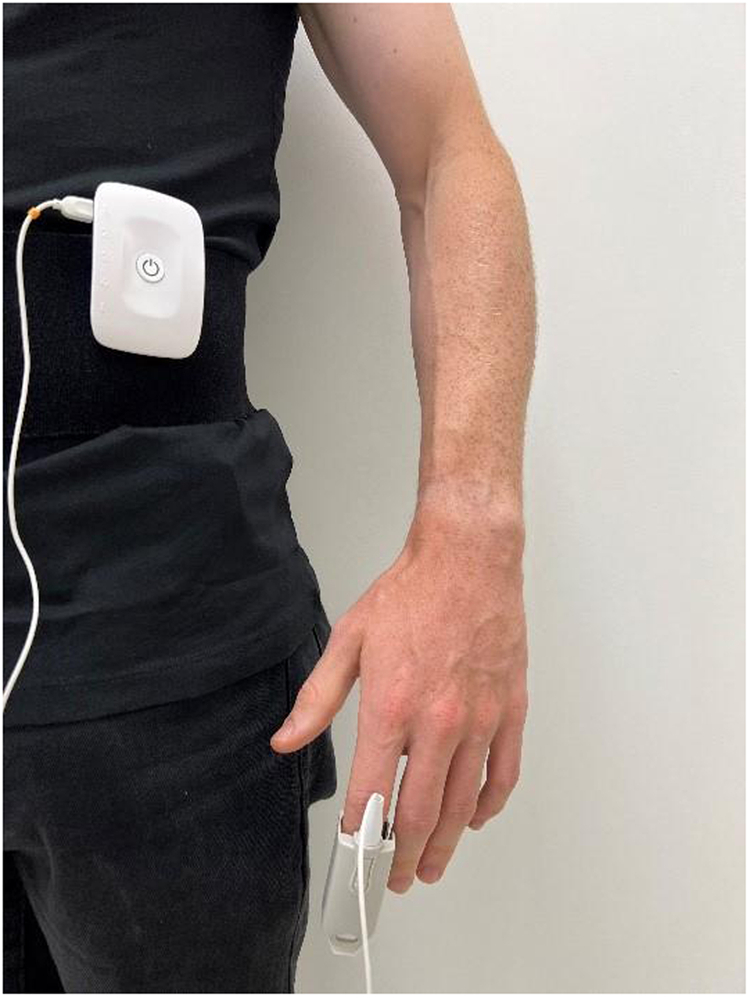
PPG sensor placement.

PPG is a method of measuring volumetric changes in blood circulation that employs a light source and a photodetector on the skin’s surface^65,66^. During shoulder IR and ER exercises, a PPG (Biosignals Plux, Portugal) (https://www.pluxbiosignals.com/products/fnirs-pioneer) wearable wireless sensor hub (45 g, dimension 54 × 85 × 10 mm) was clipped around the waist, while an optical non-invasive blood volume pulse sensor clip was placed on the non-dominant index finger to measure PPG signals with a 200 Hz sampling frequency (Fig. 1). During the tests, participants were asked to keep their non-dominant hand stable on the side of their body. PPG sensor placement is shown in Figs. 2, 3.

### Borg RPE scale measurement

The Borg’s RPE scale was used at 10-second intervals during the shoulder IR and ER exercises. The RPE scale is linear, ranges from 6 to 20 and includes descriptions ranging from ‘No effort at all’ to ‘Maximum effort’^67^. It is a reliable measure for monitoring the collective feedback of physiological, psychological, and situational factors. It allows the individuals to assess how easy or difficult a task is and how tired the participants feel when performing tasks^68^. The measurement was continued until the participants reached maximum exertion (level 20) and could not continue the exercise. The scale was positioned at eye level in front of the participants and monitored throughout each exercise. Participants were verbally encouraged to continue to exercise until they were exhausted and could not continue.

### KSS measurement

KSS is a popular method for assessing subjective sleepiness or alertness at specific moment in time^40^. The KSS assesses various dimensions of an individual’s subjective encounter with wakefulness, alertness, and fatigue^69,70^. Although the primary purpose of the KSS is to evaluate sleepiness, it is often employed simultaneously or alternatively with fatigue assessments, reflecting the close association between these two distinct concepts^71,72^. Additionally, KSS has been compared with electroencephalogram and behavioral variables, demonstrating its high validity in assessing sleepiness^40^. Therefore, we included the KSS measurements during the fatigue protocol to be used in correlation analyses with the objective data coming from the wearable sensors. The nine steps of the KSS scale range from ‘extremely alert’ to ‘very sleepy, great effort to keep awake, fighting sleep’. The test was administrated at the beginning and end of the study to assess the impact of the exercises on the participants’ levels of sleepiness and alertness^41^.

### Data processing

During the data acquisition, IMU and EMG raw data were synchronized by a trigger box and then saved into.cvs and.emt file formats, respectively. The PPG raw data were not synchronized with the aforementioned signals and are stored in a.txt file. Then, data were imported and processed using Python 3.8 (Python Software Foundation, Delaware, US). A single directory titled ‘EMG, IMU, and PPG data’ was generated, containing comprehensive information regarding IMU, PPG, and EMG. An elaborate description of this folder is provided in the section ‘Data records’.

## Data Records

Data are archived in the repository available at https://zenodo.org/record/8415066^73^. The dataset contains data from 34 individuals, including demographic and anthropometric information, MVIC force, Borg RPE Scale, KSS, EMG, IMU, and PPG data. Demographic and anthropometric information data were reported within a folder named ‘Demographic and anthropometric data’, including separate.csv files for body composition, breadth, circumference, demographic, depth, and length. Besides this, MVIC force, Borg RPE scale, and KSS data are housed in separate folders called ‘MVIC force data’, ‘Borg data’, and ‘KSS data’, respectively. Additionally, EMG, IMU, and PPG data are stored in a single folder named ‘EMG, IMU, and PPG data’.

### Demographic and anthropometric data

The demographic and anthropometric measurements of the participants are provided in a folder named ‘Demographic and anthropometric data’. This folder comprises separate files for body composition, breadth, circumference, demographic, depth, and length. The files are named ‘body_composition.csv’, ‘breadth.csv’, ‘circumference.csv’, ‘demographic.csv’, ‘depth.csv’ and ‘length.csv’.

In the ‘body_composition.csv’ file, the body weight of each participant is labelled as ‘mass (kg)’, the body fat ratio is labelled as ‘body fat%’, the skeletal muscle mass is labelled as ‘muscle%’, the visceral fat is indicated as ‘visceral fat’, and the body mass index values are provided as ‘BMI (kg/m²)’.

In the ‘breadth.csv’ file, the following measurements are recorded for each participant: upper arm middle point breadth as ‘upper_arm_mid_b_(cm)’, upper arm distal breadth as ‘upper_arm_ditsal_b_(cm)’, upper arm proximal breadth as ‘upper_arm_proximal_b_(cm)’, forearm middle point breadth as ‘forearm_mid_b_(cm)’, forearm distal breadth as ‘forearm_ditsal_b_(cm)’, forearm proximal breadth as ‘forearm_proximal_b_(cm)’, and hand breadth ‘hand_b_(cm)’.

In the ‘circumference.csv’ file, the following measurements are recorded for each participant: upper arm middle point circumference as ‘upper_arm_mid_c_(cm)’, upper arm tense circumference as ‘upper_arm_tense _c_(cm)’, upper arm distal circumference as ‘upper_arm_distal_c_(cm)’, upper arm proximal circumference as ‘upper_arm_proximal_c_(cm)’, forearm middle point circumference as ‘forearm_mid_c_(cm)’, forearm distal circumference as ‘forearm_distal_c_(cm)’, forearm proximal circumference as ‘forearm_proximal_c_(cm)’, and hand circumference as ‘hand_c_(cm)’.

On the ‘demographic.csv’ file, the date of the measurements (dd/mm/aaaa) for each participant is labelled as ‘date’, the time of the measurements (24-hour clock) is ‘time’, the order of the exercises is indicated by ‘group’, the age of the participants is ‘age’, their gender and height is noted as ‘sex’ and ‘height’, respectively, their dominant hand is ‘dominant_hand’, information about the type of exercises they engage in is described as ‘what_kind_of_exercise_do_you_participate_in?’, and the frequency with which they perform these exercises during the week is provided as ‘how_often_do_you_exercise_per_week?’.

In the ‘length.csv’ file, the upper arm length of each participant is labelled as ‘upperarm_l_(cm)’, the forearm length is ‘forearm_l_(cm)’, the hand length is ‘hand_l_(cm)’, and the palmar length is indicated as ‘palmar_l_(cm)’.

In the ‘depth.csv’ file, the following measurements are recorded for each participant: upper arm middle point depth as ‘upper_arm_mid_d_(cm)’, upper arm distal depth as ‘upper_arm_ditsal_d_(cm)’, upper arm proximal depth as ‘upper_arm_proximal_d_(cm)’, forearm middle point depth as ‘forearm_mid_d_(cm)’, forearm distal depth as ‘forearm_ditsal_d_(cm)’, forearm proximal depth as ‘forearm_proximal_d_(cm)’, and hand depth ‘hand_d_(cm)’. Anthropometric measurements are provided in Table 2.

**Table 2:** Demographic and anthropometric information.

	Mean ± SD		Mean ± SD
Subject	34	FD circumference (cm)	17.01 ± 1.62
Age (years)	26 ± 3.98	FP circumference (cm)	26.37 ± 3.04
Height (cm)	174.97 ± 9.83	H circumference (cm)	21.40 ± 2.01
Body weight (kg)	74.38 ± 18.24	UAM breadth (cm)	8.57 ± 1.26
BMI (kg/m^2^)	23.99 ± 3.82	UAD breadth (cm)	7.28 ± 1.27
Body fat (%)	25.03 ± 7.07	UAP breadth (cm)	9.04 ± 1.38
Skeletal muscle (%)	34.76 ± 5.74	FM breadth (cm)	7.16 ± 0.86
Visceral fat	5.97 ± 3.36	FD breadth (cm)	5.66 ± 0.52
Upper arm length (cm)	36.96 ± 2.04	FP breadth (cm)	8.79 ± 1.08
Forearm length (cm)	27.11 ± 1.80	H breadth (cm)	8.37 ± 0.81
Hand length (cm)	18.85 ± 1.24	UAM depth (cm)	8.32 ± 1.06
Palmar length (cm)	10.91 ± 0.91	UAD depth (cm)	7.68 ± 1.18
UAM circumference (cm)	29.49 ± 3.45	UAP depth (cm)	9.26 ± 1.30
UAT circumference (cm)	31.93 ± 3.88	FM depth (cm)	8.07 ± 0.98
UAD circumference (cm)	25.94 ± 2.84	FD depth (cm)	4.12 ± 0.48
UAP circumference (cm)	31.14 ± 3.83	FP depth (cm)	8.39 ± 1.06
FM circumference (cm)	26.61 ± 2.84	H depth (cm)	3.43 ± 0.43

### MVIC force data

The MVIC force information is stored in a folder named ‘MVIC force data’, within a file called ‘MVIC_force_data.csv’. The file comprises shoulder IR MVIC force first measurement, labelled as ‘IR_MVIC_1_(N)’, shoulder IR MVIC force second measurement, labelled as ‘IR_MVIC_2_ (N)’, mean of first and second shoulder IR MVIC force, labelled ‘IR_MVIC_mean_(N)’, shoulder ER MVIC force first measurement, labelled as ‘ER_MVIC_1_(N)’, shoulder ER MVIC force second measurement, labelled as ‘ER_MVIC_2_ (N)’, and mean of first and second shoulder ER MVIC force, labelled as ‘ER_MVIC_mean_(N)’. Descriptive statistics for the participants’ mean, minimum, and maximum MVIC force for shoulder IR and ER are given in Table 3.

**Table 3:** MVIC force information.

	Male			Female		
	Mean ± SD	Min	Max	Mean ± SD	Min	Max
IR MVIC mean (N)	102.22 ± 23.26	68.75	175	62.16 ± 8.96	50	77.5
ER MVIC mean (N)	88.52 ± 13.25	67.50	117.5	53.75 ± 9.19	35	70

### Borg RPE scale data

The Borg RPE scale values of each subject during each exercise are given in a folder named ‘Borg data’, inside a file called ‘borg_data.csv’. The Borg RPE scale values of each subject during each exercise are given in the file named ‘borg_data.csv’. The ‘task_order’ column was created to provide the sequence and load of the tasks. To ensure a clear understanding for the reader, the task sequence does not correspond to the order in which the subject executed them (group one, two, and three); instead, it maintains an unvarying order. The shoulder IR exercise performed with a load in the 30–40% range of MVIC force is named ‘task1_35i’, the shoulder IR exercise performed with a load in the 40–50% range of MVIC force is named ‘task2_45i’, and the shoulder IR exercise performed with a load in the 50–60% range of MVIC force is named ‘task3_55i’; while the shoulder ER exercise performed with a load in the 30–40% range of MVIC force is named ‘task4_35e’, the shoulder ER exercise performed with a load in the 40–50% range of MVIC force is named ‘task5_45e’, the shoulder ER exercise performed with a load in the 50–60% range of MVIC force is named ‘task6_55e’.

The level of difficulty perceived by the participants before the exercises is provided in the ‘before_task’ column, while the participants’ difficulty level at 10 seconds after the commencement of the exercise is indicated as ‘10_sec’. Progressing similarly at 20 seconds the label is ‘20_sec’, and so forth until the column named ‘250_sec’ Afterward, the column in which the participants reached the level of 20 on the Borg RPE scale was given separately as ‘end_of_trial’. Finally, the duration of each exercise is specified as ‘length_of_trial_(sec)’. Table 4 provides the descriptive statistics for the participants, including the mean, minimum, and maximum duration times in seconds for Borg RPE.

**Table 4:** Borg RPE scale information.

	Male			Female		
	Mean ± SD	Min	Max	Mean ± SD	Min	Max
IR 30–40% (sec)	117.91 ± 29.52	67	180	112.00 ± 56.86	53	250
IR 40–50% (sec)	79.74 ± 25.84	46	152	90.73 ± 50.02	50	202
IR 50–60% (sec)	70.78 ± 27.57	30	125	76.64 ± 45.74	34	210
ER 30–40% (sec)	59.39 ± 10.44	40	81	67.09 ± 18.79	40	108
ER 40–50% (sec)	50.26 ± 9.29	30	67	55.27 ± 11.82	42	78
ER 50–60% (sec)	42.70 ± 10.27	18	60	46.00 ± 12.58	33	70

### KSS data

Information regarding the KSS data of each subject is provided in a folder named ‘KSS data’, in a file called ‘KSS.csv’.Information regarding the KSS data of each subject is provided in an file named ‘KSS_data.csv’. KSS scores before the exercises are labelled as ‘KSS_before’ and the KSS scores at the very end of the exercises are labelled as ‘KSS_after’. Table 5 presents the summary of the participants’ KSS sleepiness levels, detailing the mean, min, max scores, and p-value between KSS before and after.

**Table 5:** KSS information.

	Male				Female			
	Mean ± SD	p-value	Min	Max	Mean ± SD	p-value	Min	Max
KSS before	4.04 ± 1.74	0.33	1	7	3.82 ± 1.54	0.30	2	6
KSS after	4.52 ± 1.56		1	7	4.55 ± 1.63		2	7

### Wearable data

**Fig. 4:**
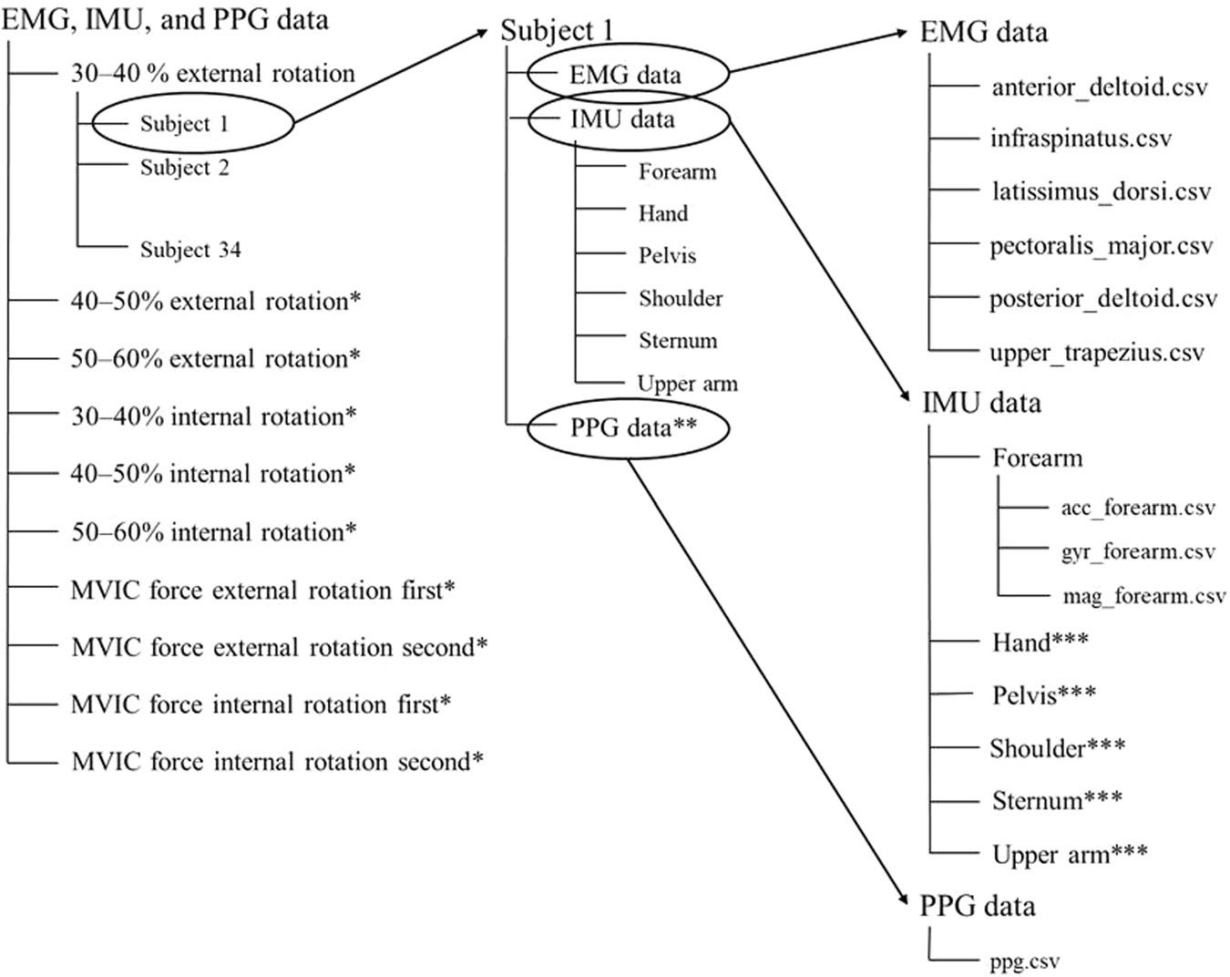
Structure of the single folder where EMG, IMU and PPG data are stored. * Each exercise includes information from the 34 participants. ** PPG data are not included in the exercises related with the MVIC force. *** Each body position includes the three.csv file associated with acc, gyr, and mag.

**Fig. 5:**
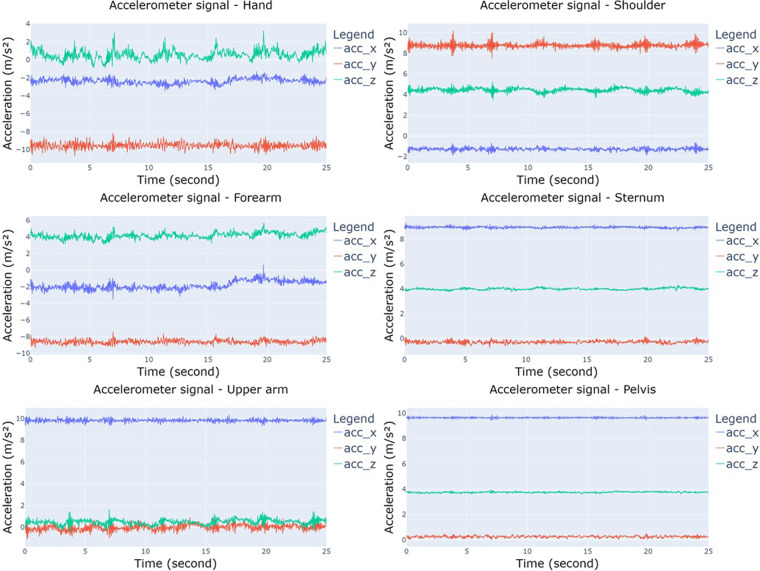
Accelerometer data from 30–40% internal rotation for hand, forearm, upper arm, shoulder, sternum, and pelvis.

**Fig. 6:**
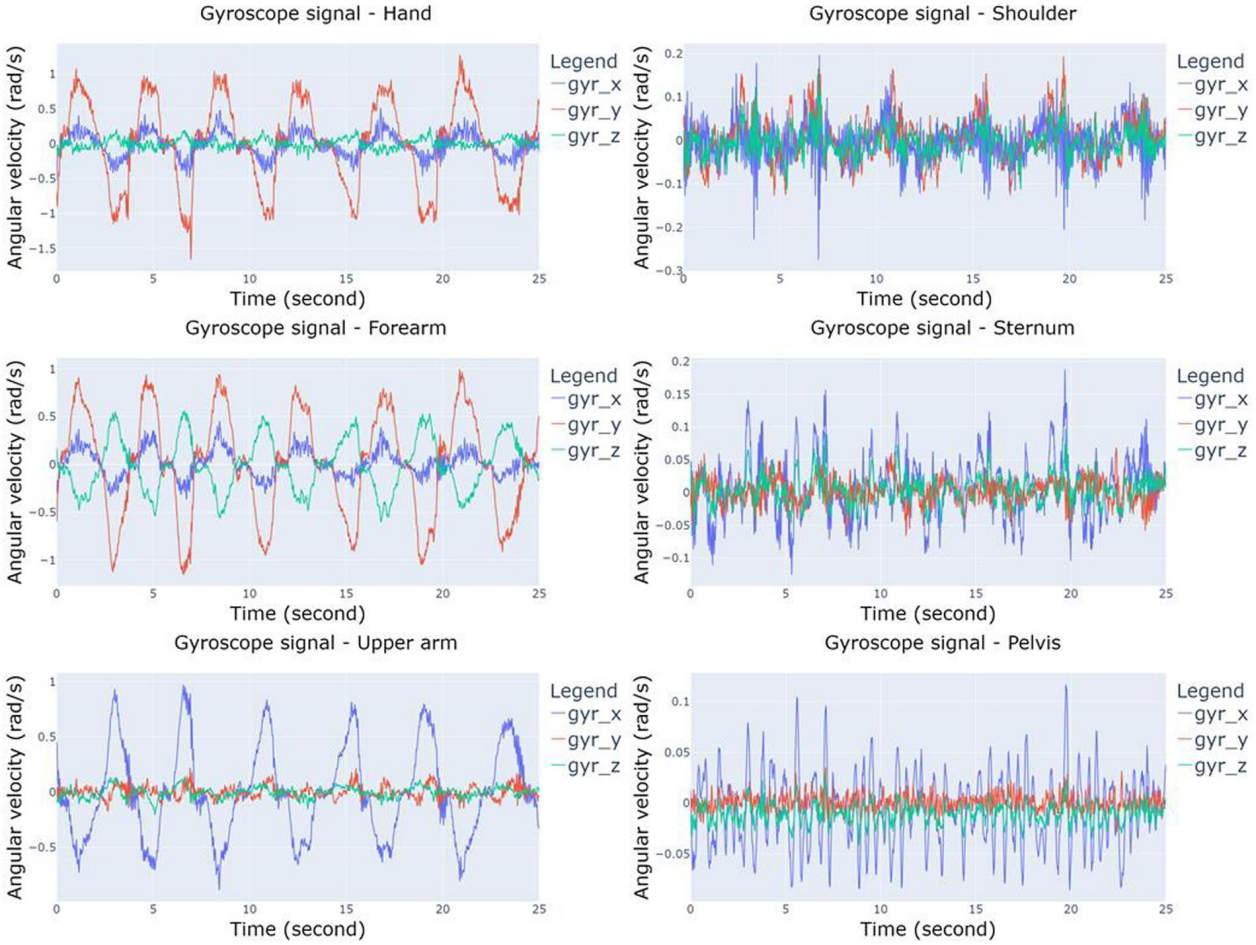
Gyroscope data from 30–40% internal rotation for hand, forearm, upper arm, shoulder, sternum, and pelvis.

**Fig. 7:**
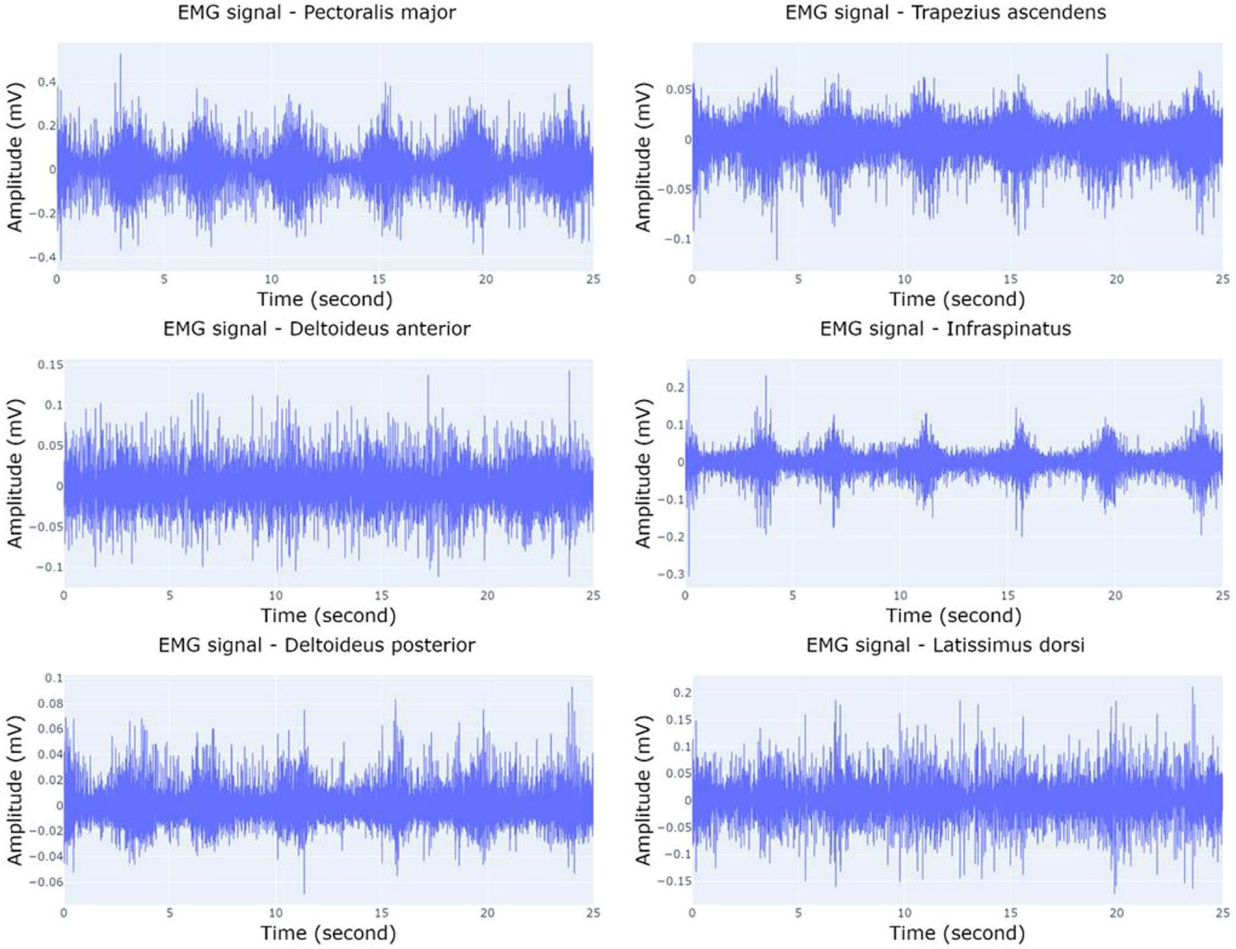
EMG data from 30–40% internal rotation for pectoralis major, deltoideus anterior, deltoideus posterior, trapezius ascendens, infraspinatus, and latissimus dorsi.

**Fig. 8:**
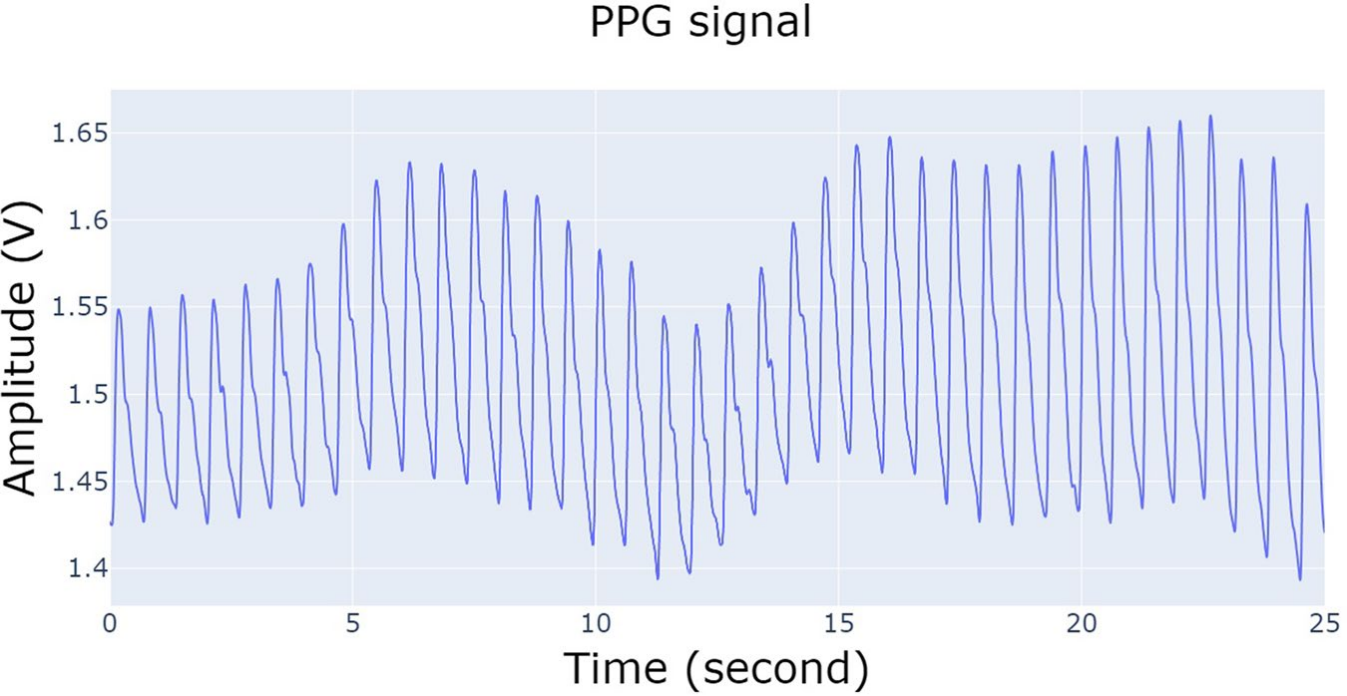
PPG data from 30–40% internal rotation.

Figure 4 illustrates the structure of the single folder where EMG, IMU, and PPG data are stored. The main folder contains 10 subfolders representing the different exercises, named ‘30–40% external rotation’, ‘40–50% external rotation’, ‘50–60% external rotation’, ‘30–40% internal rotation’, ‘40–50% internal rotation’, ‘50–60% internal rotation’, ‘MVIC force external rotation first’, ‘MVIC force external rotation second’, ‘MVIC force internal rotation first’, and ‘MVIC force external rotation first’, respectively. Every exercise includes information for the 34 subjects, and within each subject’s directory, there exist EMG, IMU, and PPG data labelled as ‘EMG data’, ‘IMU data’, and ‘PPG data’, respectively. The ‘EMG data’ folders comprise six files named ‘anterior_deltoid.csv’, ‘infraspinatus.csv’, ‘latissimus_dorsi.csv ‘, ‘pectoralis_major.csv ‘, ‘posterior_deltoid.csv ‘, and ‘upper_trapezius.csv ‘ and contain measurements in millivolts (mV); while ‘PPG data’ folders have one single file titled ‘ppg.csv’ with Volt as metric. PPG data are not included in the exercises related to the MVIC force. Regarding the data derived from IMU sensors, every participant includes a directory denoted as ‘IMU data’ that comprises six subdirectories corresponding to the forearm, hand, pelvis, shoulder, sternum, and upper arm. Within each of these, there exist three.csv files labelled as ‘acc_x,’ ‘gyr_x,’ and ‘mag_x,’ where x represents the specific body part (forearm, hand, pelvis, shoulder, sternum, or upper arm), depending on the relative folder. ‘acc_x’ data are expressed in meters per second squared (m/s²), ‘gyr_x’ data in radians per second (rad/s), and ‘mag_x’ in arbitrary units (a.u.). Figures 5–8 depict a 25-second recording from the ‘30–40% internal rotation’ registration, corresponding to acc, gyr, ECG, and PPG data, respectively.

## Technical Validation

### Sensor placement

The same researcher accurately and consistently positioned the EMG and IMU sensors on each participant, adhering to the reference areas specified in the literature and by the manufacturer’s guidelines. It was ensured that the sensors were positioned in the same position for each participant and correctly aligned. Before and after each measurement, IMU sensor straps were checked for looseness and stability, and the EMG electrodes were inspected for surface adhesion to the muscle. Furthermore, prior to every exercise, the system’s acquisition software was used to manually confirm the signal accuracy of each IMU sensor. For each individual measurement, excluding those associated with the MVIC force, the PPG signal was obtained from the participants’ index fingers. This was achieved by meticulously attaching the finger clip sensor to ensure accurate data collection.

### Comparison with published datasets

There are several available datasets for fatigue measurements using wearable sensors. Kalanadhabhatta *et al*. (2022) created an extensive dataset consisting of PPG, electroencephalography (EEG), electrocardiography (ECG), electrodermal activity (EDA), acc, gyr, and skin temperature data to improve the understanding of mental fatigue and exhaustion in daily life^44^. In a different dataset, the authors aimed to understand the relationship between self-reported fatigue and sensor data through machine learning approaches. For this purpose, a fatigue questionnaire and a multi-sensor wearable device were used which included galvanic skin response electrodes, acc, barometers, and photo and temperature sensors^42^. Another study published a dataset to predict fatigue with machine learning models in the biceps muscle using wearable IMU sensors during biceps curl exercise^47^. Papakostas *et al*. (2009) published a dataset, consisting of 19 participants, designed for machine learning-based experiments to evaluate the effects of cognitive fatigue on human performance using EEG, real-time self-reports on cognitive fatigue, facial key points, and details regarding the performance (such as the number of errors)^45^. Li and Zhang^43^ published a driving fatigue dataset consisting of the heart rate and facial features of 20 drivers^43^.

Unlike these datasets, our study provides information about fatigue during dynamic shoulder rotation movements with different loads. Furthermore, our research contains EMG sensor data which can give valuable information to indicate muscle fatigue^74^. Jaiswal *et al*.^46^ published a large dataset in their study to evaluate physical and cognitive fatigue, employing wearable sensors and advanced machine learning techniques during treadmill running. The dataset consisted of ECG, EMG, EEG, and EDA data from 32 healthy participants and their self-reported fatigue states^46^. This data collection, unlike the one presented in this paper, assesses fatigue during the treadmill run and provides information on overall fatigue. By evaluating fatigue towards target muscle groups during specific shoulder IR and ER motions, we want to detect fatigue during tasks when the upper extremity is primarily utilised. In addition, our data collection contains a wide range of anthropometric measurement data, allowing fatigue to be potentially modelled based on physical characteristics or in combination with machine learning methods. Maman *et al*.^25^ used wearable IMU and heart rate sensors to model the fatigue of eight participants during simulated manufacturing tasks^25^. Again, this study’s dataset differs from ours because it only measures fatigue during specific industry-related tasks. However, the tasks selected for our study include shoulder IR and ER movements that can be applied to a variety of shoulder-dominant contexts including sports science, physiotherapy, and industry. The dataset of this study also differs from ours in that it contains fewer participants. The number of volunteers is an important technical component for database selection because a large quantity of data is required for machine learning model training^42^. The size of 34 participants is comparable to other dataset articles in the literature that propose building AI-based algorithms, such as machine learning models, with wearable sensors^42,75^.

### Limitations

Initially, the dataset comprised 40 subjects; however, the pool was decreased to 34 participants. This reduction was required by the discovery of unreliable data corresponding to six individuals. Of these, five were omitted due to inaccurate PPG signal, while a single participant was excluded owing to invalid magnetometer readings. Additionally, the acc, gyr, and mag files located within the directory EMG, IMU, and PPG data\30–40% internal rotation\Subject 3\IMU data\Shoulder are corrupted and cannot be used.

The signal quality for the PPG sensor was evaluated in line with the recommendations of previous studies^76^ and manufacturer guidelines^77^. Due to noisy and unclear signals, five participants had to be excluded from the study. The conclusion derived from the weak signal quality is that it correlates with variations in skin tone. This phenomenon is consistent with research findings indicating that signal quality decreases in individuals with darker skin tones^78^. The absorption of green light by melanin in those with darker skin tones limits light penetration into the subcutaneous region where blood is present^79^. In addition, the PPG signals were not hardware or firmware synchronized with the data provided by the EMG and IMU sensors. The alignment was accomplished by manually halting all data recordings in unison and, subsequently, synchronizing them retroactively. Yet, this assumption is constrained by the absence of a hardware or firmware-based approach.

Regarding the data from the inertial sensors, the magnetometer data from IMU sensors may be sensitive to an alteration in magnetic fields. Because of this, the testing location was set up in accordance with the guidelines from previous studies^80^ and remained consistent for every participant. Due to magnetic field interference, one participant had to be excluded from the study.

This dataset is limited by data collected only from healthy young individuals. Likewise, fatigue could be examined across various age categories. For example, Yoon *et al*.^81^ reported that for a low-force task, EMG activity during fatigue contraction was higher in older people^81^. In another study, variations related to age were found to influence postural kinematics and joint kinetics during repetitive lifting^82^. Further data should be collected in future works for monitoring shoulder joint fatigue in upper extremity athletes, industrial employees, and physical therapy patients with shoulder discomfort.

Lastly, the present work focuses on physical fatigue measurements during dynamic shoulder rotation movements using low-cost movement and EMG sensors. However, as shown in the literature, this is only one approach to the problem of fatigue estimation and several other technologies and contexts may have been taken into consideration. For instance, Yu *et al*.^83^ focused on construction workers using biomechanical analysis carried out on 3D model data gathered from computer vision systems^83^, Papoutsakis *et al*.^84^ looked instead at passive cameras sensors in a real manufacturing workplace^84^, while other studies investigated the impact of cardiorespiratory and thermoregulatory measurements in firefighters or healthy subjects^85,86^. Finally, even wearable pressure insoles are promising tools to this purpose in laboratory settings^87^. Hence, future research should be focusing towards the publication of data repositories of physical and cognitive fatigue also taking into consideration a broader range of technologies and real-world applications.

## Usage Notes

Prior studies have demonstrated the effectiveness of IMUs, EMG, and PPG wearable devices in accurately assessing fatigue, with features extracted to train machine learning models^24,25,49,88,89^. This study provides information on the onset of fatigue during complex shoulder movements with different loads. It provides comprehensive anthropometric measurements, allowing the parametrisation of biomechanical models based on individual differences. This approach can be applied to workers under various workloads at work so to design AI-based or biomechanical-based models (or a combination) able to recognize the onset of fatigue. Workers can be alerted to high fatigue levels and encouraged to take proper breaks using real-time wearable sensor inputs (e.g., sound alert on phone or wristwatch). Besides that, exceeding a certain threshold in exercise intensity can pose risks to the human body. As a result, ensuring athletes’ safety and improving their competitive performance necessitates precise regulation and fine-tuning of the exercise load. Deep learning techniques can be used to forecast the state of exercise fatigue in the human body by means of data from wearable sensors^90^. Physical activity is also essential in improving individuals’ health in various rehabilitation settings, and careful regulation of exercise intensity is important^91^. These intensity levels can be monitored using a set of wearables proposed in this work, allowing exercise intensity to be precisely modulated during treatment sessions^22^. Finally, suitable AI-based approach can provide real-time and personalized fatigue management during the rehabilitation process^92^.

To facilitate the widespread use of the dataset for fatigue detection algorithms, the raw data is provided in easy-to-access.csv format.

## Data Availability

We supply the raw data in.csv files and have not used any ad-hoc code to process them.
